# Label-free electrochemical immunosensor as a reliable point-of-care device for the detection of Interleukin-6 in serum samples from patients with psoriasis

**DOI:** 10.3389/fchem.2023.1251360

**Published:** 2023-11-10

**Authors:** Rocco Cancelliere, Terenzio Cosio, Elena Campione, Martina Corvino, Maria Pia D’Amico, Laura Micheli, Emanuela Signori, Giorgio Contini

**Affiliations:** ^1^ Department of Chemical Science and Technologies, University of Rome Tor Vergata, Roma, Italy; ^2^ Dermatology Unit, Department of Systems Medicine, University of Rome Tor Vergata, Roma, Italy; ^3^ Department of Experimental Medicine, University of Rome Tor Vergata, Roma, Italy; ^4^ Istituto di Farmacologia Traslazionale-CNR (IFT-CNR), Roma, Italy; ^5^ Istituto di Struttura Della Materia-CNR (ISM-CNR), Roma, Italy; ^6^ Department of Physics, University of Rome Tor Vergata, Roma, Italy

**Keywords:** interleukin-6, sandwich-based immunosensor, biochar, serum samples, psoriasis, point-ofcare

## Abstract

Interleukin-6 (IL-6) plays a crucial role in autoimmunity and chronic inflammation. This study aims to develop a low-cost, simple-to-manufacture, and user-friendly label-free electrochemical point-of-care device for the rapid detection of IL-6 in patients with psoriasis. Precisely, a sandwich-based format immunosensor was developed using two primary antibodies (mAb-IL6 clone-5 and clone-7) and screen-printed electrodes modified with an inexpensive recycling electrochemical enhancing material, called biochar. mAb-IL6 clone-5 was used as a covalently immobilized capture bioreceptor on modified electrodes, and mAb-IL6 clone-7 was used to recognize the immunocomplex (Anti-IL6 clone-5 and IL-6) and form the sandwich. Cyclic voltammetry (CV) and electrochemical impedance spectroscopy (EIS) were used to conduct electrochemical characterization of the layer-by-layer assembly of the immunosensor, while square wave voltammetry (SWV) was used to perform the sensing. The developed immunosensor demonstrated robust analytical performance in buffer solution, with a wide linear range (LR) by varying from 2 to 250 pg/mL, a good limit of detection (LOD) of 0.78 pg/mL and reproducibility (RSD<7%). In addition, a spectrophotometric ELISA kit was employed to validate the results obtained with the label-free device by analyzing twenty-five serum samples from control and patients affected by psoriasis. A strong correlation in terms of pg/mL concentration of IL-6 was found comparing the two methods, with the advantage for our label-free biosensor of an ease use and a quicker detection time. Based on IL-6 levels, the proposed immunosensor is a dependable, non-invasive screening device capable of predicting disease onset, progression, and treatment efficacy.

## 1 Introduction

Over the centuries infectious and inflammatory diseases have significantly impacted global health. Nowadays, the recent pandemic COVID-19 highlighted the importance of developing novel point-of-care (POC) diagnostics to improve the timely recognition and therapeutic monitoring of these diseases. POC devices provide various advantages over labour- and time-intensive traditional diagnostic methods, such as faster screening, improved sensitivity and specificity, reduced cost, good efficiency, and the capacity for on-site detection. Label-free biosensors have emerged also as powerful analytical devices for detecting and quantifying biomolecules in various fields, *e.g.*, environmental monitoring. They can detect a wide range of analytes, including proteins, nucleic acids, and small molecules, making them versatile tools for diverse applications (Cooper, 2009; Schöning and Poghossian, 2018; Pasquarelli, 2021; Samuel and Rao, 2022). These biosensors offer several advantages over traditional immunoassays such as the Enzyme-Linked Immunosorbent Assay (ELISA). ELISA test is a powerful technique used to detect reveal and quantify specific proteins or antigens in a sample. It relies on the specific binding between an immobilized capture antibody and the target analyte, followed by the detection of the bound analyte using an enzyme-labelled detection antibody. A modified version of the competitive ELISA is the sandwich ELISA. The key difference between these two assays lies in the number of antibodies involved and their binding patterns. In contrast to conventional competitive ELISA, which uses a single antibody for both capture and detection, sandwich ELISA employs two antibodies that recognise distinct antigen epitopes. This permits increased specificity and sensitivity, as the target antigen is sandwiched between two antibodies, minimising interference from other molecules in the sample (Hnasko and McGarvey, 2015; Kováčiková et al., 2018). On this basis, sandwich label-free biosensors have been realized, providing the possibility of multiplexing, and allowing the simultaneous detection of different analytes within a single sample. By utilizing diverse capture and detection elements, specific to different biomolecules, these biosensors enable the determination of a panel of biomarkers or the analysis of complex biological samples in a high-throughput manner, with enhanced detection limits and accuracy. They can be also used to identify and determine inflammatory biomarkers such as Interleukin-6 (IL-6). IL-6 is a cytokine that plays a critical role in the regulation of immune responses and inflammatory processes within the human body. It is produced in response to infections, tissue damage, and other inflammatory stimuli. The dysregulation of IL-6 signalling is particularly implicated in the pathogenesis of numerous inflammatory pathologies, ranging from chronic inflammatory diseases to autoimmune disorders. Elevated levels of IL-6 are observed in rheumatoid arthritis, *systemic lupus erythematosus*, inflammatory bowel disease and sepsis, highlighting the role of this cytokine as a key mediator of inflammation. In these conditions, excessive IL-6 production contributes to the perpetuation of the inflammatory response, leading to tissue damage and disease progression (Tanaka and Kishimoto, 2012; Scheller et al., 2014; Tanaka et al., 2014; Hunter and Jones, 2015). Among autoimmune diseases, psoriasis (PsO) is a chronic inflammatory skin pathology characterized by red, scaly patches that can cause significant physical and psychological distress to affected individuals (Caputo et al., 2020). The accurate and timely measurement of biomarkers associated with PsO, such as IL-6, is essential for disease diagnosis, monitoring, and treatment evaluation (Monteleone et al., 2011; Rendon and Schäkel, 2019). Elevated levels of IL-6 have been observed in both serum and skin lesions of PsO patients, correlating with a severe illness (Kutsuna et al., 2022). In this regard, Grossman (Grossman et al., 1989) and his colleagues demonstrated for the first time in 1989 that IL-6 could directly contribute to the epidermal hyperplasia observed in psoriatic epithelium influencing the function of dermal inflammatory cells. They found elevated levels of IL-6 in the plasma of patients with active PsO (mean: 3 ng/mL), demonstrating a direct correlation between IL-6 expression and keratinocytes proliferation. In PsO, IL-6 contributes directly to the differentiation of pathogenic Th17 cells (Zhou et al., 2007), which are associated with the initiation of autoimmunity and inflammation. The cells, recently identified in cell suspensions from lesional psoriatic skin (Lowes et al., 2008), were reduced following clinical improvement (Zaba et al., 2007) and implicated in disease pathogenesis due to the marked clinical effectiveness of anti-IL-12/23p40, which leads to dramatic reductions of IL-23p19 but not IL-12p35 (Toichi et al., 2006). Therefore, accurate and timely detection of IL-6 levels is essential for disease monitoring, evaluation of treatment efficacy, and identification of possible adverse effects related to the administration of therapies (Monteleone et al., 2011; Mohd Noor et al., 2022). In this context, label-free electrochemical immunosensors have emerged as promising POC devices for IL-6 detection, offering a valid alternative to traditional techniques which require a in-presence sample processing and high employment of reagents and time (Oh et al., 2021). Our group has developed a direct label-free immunosensor for IL-6 detection in human serum and blood (Cancelliere et al., 2022a). In that research, bare screen-printed electrodes (SPEs) were modified with a pyrolytic carbonaceous material derived from biomasses, which has the unique ability to function as both an electrochemical enhancing material and a substrate for protein binding (anchoring system) (Cancelliere et al., 2022b). This platform, despite exhibiting promising sensitivity and reproducibility, presented selectivity that could be improved.

In this aim, here a new sandwich-format label-free electrochemical immunosensor based on two primary antibodies, mAb-IL-6 clone 5 (immobilised on screen-printed electrodes) and mAb-IL-6 clone 7 (for the sandwich formation and immuno-complex recognition), was developed. These immunosensing platforms were initially tested in buffer and spiked serum solution, showing improved sensitivity (limit of detection, LOD), linear range (LR) and comparable reproducibility in comparison with the direct label-free immunosensor. Its selectivity was examined by analysing the sandwich-based label-free immunosensor’s cross-reactivity over several cytokines, showing always excellent results (cross-reactivity<20%) and demonstrating the device’s affordability and enhancement. Serum samples from 25 psoriasis-affected and control patients were used as a real matrix for the validation of the device.

By combining the advantages of label-free detection with the sandwich assay format, the obtained results demonstrate it is possible to achieve a real-time monitoring system with enhanced sensitivity and specificity, easy-to-use and efficient as warning POC. Currently, there is no diagnostic test for PsO. Dermatologists can typically diagnose PsO simply by examining a patient’s skin. A skin biopsy may be performed to obtain additional information, confirm the diagnosis, and rule out other possible causes of symptoms, such as eczema or cutaneous lupus (Kim et al., 2017; Kimmel and Lebwohl, 2018; Mirghani et al., 2022). In this scenario, the proposed POC is a non-invasive screening instrument that can predict PsO disease onset, progression, and potential treatment efficacy based on IL-6 levels detection.

## 2 Materials and methods

### 2.1 Reagents

All used chemicals were of analytical grade. N-hydroxysuccinimide (NHS, 98%) and N-(3-Dimethylaminopropyl)-N′-ethyl carbodiimide (EDC, ≥97%) were purchased from Sigma-Aldrich (Germany). Clone-5 and clone-7 monoclonal anti-Interleukin-6 antibodies (mAb) were purchased from Thermo Fisher Scientific (United States of America). Interleukins 1-β (IL-1-β), IL-2, IL-5, IL-6, IL-10, IL-12, Tumor Necrosis Factor α and β (TNF-α and TNF-β) and Normal Human Serum (31876) were purchased from Thermo Fisher Scientific (United States of America). Sodium bicarbonate, potassium chloride and polyvinyl alcohol (PVA) were purchased from Sigma Aldrich (Germany). Human IL-6 ELISA kit was purchased from Invitrogen (Thermo Fisher Scientific, United States). Screen-printed electrodes were in-house produced at the University of Roma Tor Vergata. The carbonaceous nanomaterial (biochar), supplied by CREA Research Center (Roma, Italy), was obtained via a pyrolytic process (Cancelliere et al., 2023b). Buffer solutions used are 0.05 M carbonate buffer, pH = 9.6 (CB); 1% PVA (w/v) solution in 0.05 M carbonate buffer, pH = 9.6 (PVA-CB); 0.05 M phosphate buffer saline +0.1 M KCl, pH = 7.4 (PBS). Serum samples from PsO patients were collected by the Tor Vergata University Hospital and donated for IL-6 detection.

### 2.2 Apparatus

PalmSens 4 Instrument was utilized for amperometry, cyclic voltammetry (CV), square wave voltammetry (SWV), and electrochemical impedance spectroscopy (EIS) investigation. Graphite SPEs produced in-house at the laboratory of the University of Roma Tor Vergata were used for the electrochemical investigation (Cancelliere et al., 2020; Di Tinno et al., 2022). Utilizing the Hielscher UP200St-Ultrasonic Transducer, biochar dispersions were produced. The agitating operation was carried out using an Orbital Shaker Platform (OSP, Grant-Bio, United Kingdom). iMark™ Microplate Absorbance Reader is used for the spectrophotometric analysis of samples.

### 2.3 Preparation of label-free electrochemical immunosensor for IL-6 detection

**FIGURE 1:**
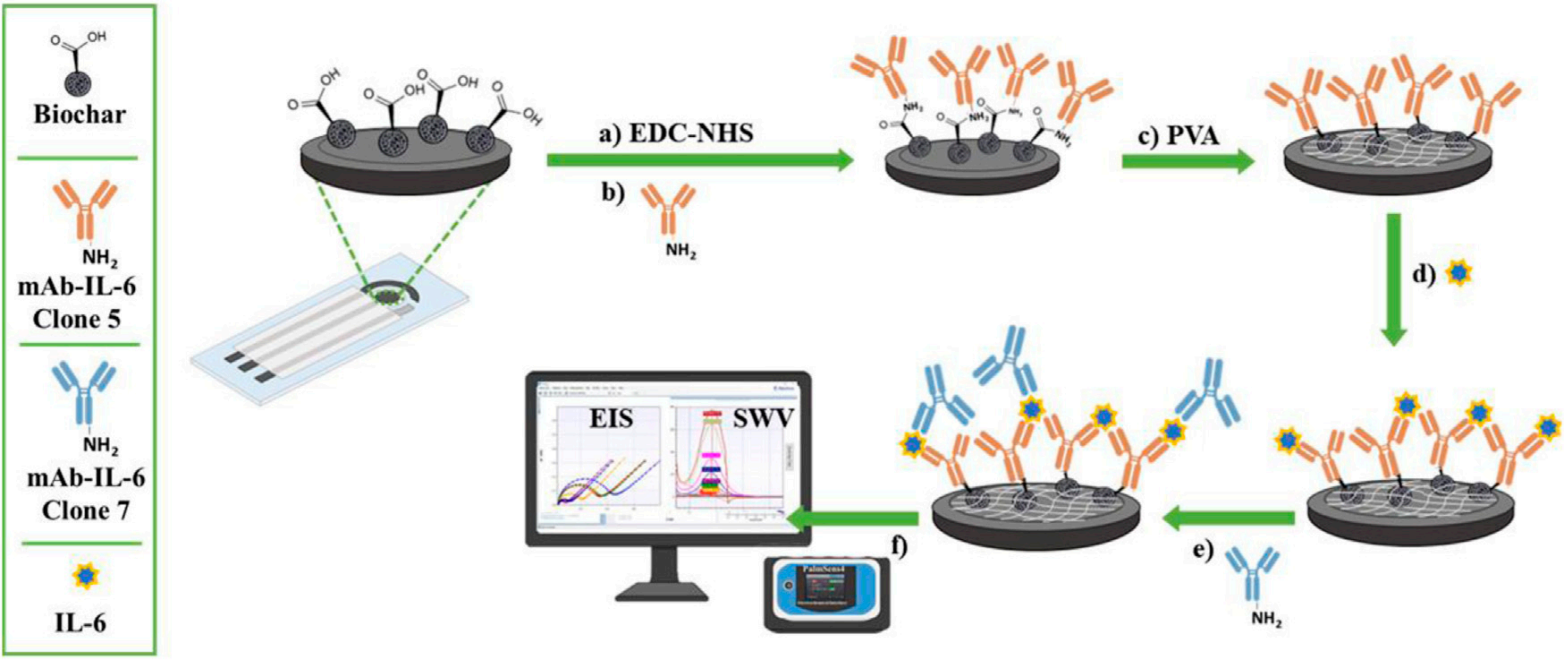
Diagrammatic representation of the fabrication procedures and detection of the IL-6 immunosensor. In **(A)** and **(B)** the carbodiimide-mediated amide coupling reaction to immobilize a specific IL-6 antibody (mAb-IL-6-Clone 5) onto biochar-modified screen-printed electrodes. In **(C)** the back-filling step using PVA and **(D)** the immunocomplex formation. In **(E)** and **(F)** the sandwich formation after adding a second primary antibody (mAb-IL-6-Clone 7) and the electrochemical detection, respectively.

In a 0.05 M PBS solution, SPEs were initially pretreated using amperometry as an analytical technique constant anodic potential of +1.7 V for 180 s 6 μL of a biochar dispersion (1 mg/mL in 1:3 v/v ethanol-water solution) prepared by an ultrasonic transducer (200 W, 26 kHz, 30 min) was drop-cast onto the treated SPE. The carboxylic groups of biochar were activated by dumping 10 µL of an EDC/NHS solution in water (1:1 w/w, freshly made by combining 0.005 M EDC and 0.005 M NHS) on the working electrode (WE) in a dark box for 20 min. Then, 6 µL of anti-Interleukin-6 Clone-5 monoclonal primary antibody (mAb IL-6c5) was drop-cast onto the WE and stored overnight at 4°C. For 15 min, 6 µL of 1% PVA in CB was incubated at room temperature (RT) to minimize the non-specific binding of other proteins’ bodies and to block the surface of WE, which remained unbound at RT. After a step of washing, 6 µL of IL-6 were added and incubated at RT for 15 min. To complete the sandwich, 5 μg/mL of anti-Interleukin-6 Clone-7 monoclonal primary antibody (mAb-IL-6c7) were subsequently added. This binding process lasted 30 min at RT, during which SPE was stirred with an orbital shaker. To remove unbound species, the electrodes were washed three times with 20 µL of distilled water (for biochar, EDC/NHS, and PVA) or PBS (for IL-6, mAb-IL-6c5 and mAb-IL-6c7 antibodies) and dried under nitrogen gas flow after each step. Using 0.01 M [Fe(CN)_6_]^4-/3-^ solution. Figure 1 reports the fabrication procedure.

### 2.4 Theory section

The heterogeneous electron transfer constant (k^0^) for the reversible electrode probe [Fe(CN)_6_]^−3/−4^ was calculated voltammetrically using Eq. 1 (Eq. 1) and Eq. 2 (Eq- 2) (Randviir and Banks, 2013; Cancelliere et al., 2021).

$$k^{0}=\varphi \sqrt{\frac{D_{0}\pi \nu F}{RT}{\left(\frac{D_{Red}}{D_{Ox}}\right)}^{\alpha }}$$ (1)

$$\varphi =\frac{\left(-0.6288+0.0021\cdot \Delta E\right)}{\left(1-0.0170\cdot \Delta E\right)}$$ (2)

where *D*_*0*_ is the average diffusion coefficient, calculated as the average of the anodic and cathodic peak current, *D*_*Ox*_ and *D*_*Red*_ are the diffusion coefficients for the ferricyanide/ferrocyanide redox reaction, respectively, *ν* is the scan rate (V/s), F is the Faraday constant (mol^−1^), *T* is the temperature (K), R is the universal gas constant (J/Kmol), α the dimensional transfer coefficient (chosen to be equal to 0.5, assuming the ratio of the anodic (Ipa) and cathodic peak current (Ipc) equal approximately to 1). The parameter φ can be obtained using the Nickolson method, using the equation Eq.2 (*ΔE* is the peak-to-peak separation).

The electron transfer constant (k^0’^) was calculated by Eq. 3 (Eq. 3) (Barsoukov, 2018; Cancelliere et al., 2021; Cancelliere et al., 2023b):

$$k^{0^{\prime }}=\frac{RT}{n^{2}F^{2}ACR_{ct}}$$ (3)

Where *n* is the number of electrons involved in the process, A is the electrode surface (cm^2^), C is the concentration of the redox couple ferro-ferricyanide (mol/L), Rct is the charge transfer resistance (Ω) and R, T, and F were previously described.

The calibration curve was obtained by plotting the IL-6 concentrations against the Ipa measured using SWV (Eq. 4). The Four Parameter Logistic (4 PL) Regression was used to fit the standard curves (Law, 1996).

$$Ipa\left(x\right)=\left[d+\frac{\left(a-d\right)}{1+{\left(\frac{x}{c}\right)}^{b}}\right]$$ (4)

where *Ipa(x)* is the anodic current (I_pa_), *a* is the minimum value that can be obtained (i.e., at 0 doses), *d* is the maximum value that can be obtained (i.e., at infinite dose), *c* is the point of inflexion (i.e., the point on the S-shaped curve halfway between a and d), and *b* is the Hill’s slope of the curve (i.e., this is related to the steepness of the curve at point c) (Law, 1996).

The LOD, Eq. 5, and the limit of quantification (LOQ), Eq. 6, were calculated as follows:

$$LOD=S_{B}+3\;S_{D}$$ (5)

$$LOQ=S_{B}+10\;S_{D}$$ (6)

where S_B_ is the signal measured in the absence of IL-6 and S_D_ is the standard deviation of the blank (10 replications) (Gustavo González and Ángeles Herrador, 2007).

The normalization of the current values was accomplished by applying the following formula, Eq. 7 (Law, 1996):

$$I\%=\left[\frac{\left(I-I_{\min}\right)}{\left(I_{\max}-I_{\min}\right)}\right]∙100$$ (7)

In which, the *I%* represent the normalized faradic current for a given IL-6 concentration, while *I*_max_ and *I*_min_ correspond to the maximum and minimum current values observed during the analysis, respectively.

The cross-reactivity experiments, investigated using the immunosensor percentage responses (% response) toward different tested cytokines (IL-1-β, IL-2, IL-5, IL-6, IL-12 and TNF-α), were obtained using the equation below (Law, 1996):

$$\%response=\left(\frac{i_{ILn}}{i_{IL-6}}\right)$$ (8)

in which *i*_*ILn*_ and *i*_*IL-6*_ are the faradic current recorded when interfering cytokines and IL-6 are incubated, respectively.

### 2.5 Study population

Twenty-five patients were consecutively enrolled in the Dermatology Unit of Roma Tor Vergata University Hospital. Men and women over the age of 18 who had a history of cutaneous PsO diagnosis were considered. Men or women under the age of 18, pregnancy, breastfeeding, systemic or topical treatments within the previous 6 months, neurological syndromes, including use of neurotropic drugs, severe renal failure, severe hepatic insufficiency, solid neoplasms or oncohematological malignancies were exclusion criteria. The blood samples were obtained at the first clinical visit (T0) before any treatment, and collected by peripheral vascular sampling using VACUETTE^®^ TUBE 8 mL CAT Serum Separator (Greiner Bio-One, Italy). The serum was stored at −20°C before analyses. All patients provided written informed consent after receiving a thorough explanation of the study’s objectives and risks. The Tor Vergata University Hospital Ethics Committee approved the study (Code 140/23).

## 3 Results and discussion

### 3.1 Assembly of IL-6 label-free immunosensor

**FIGURE 2:**
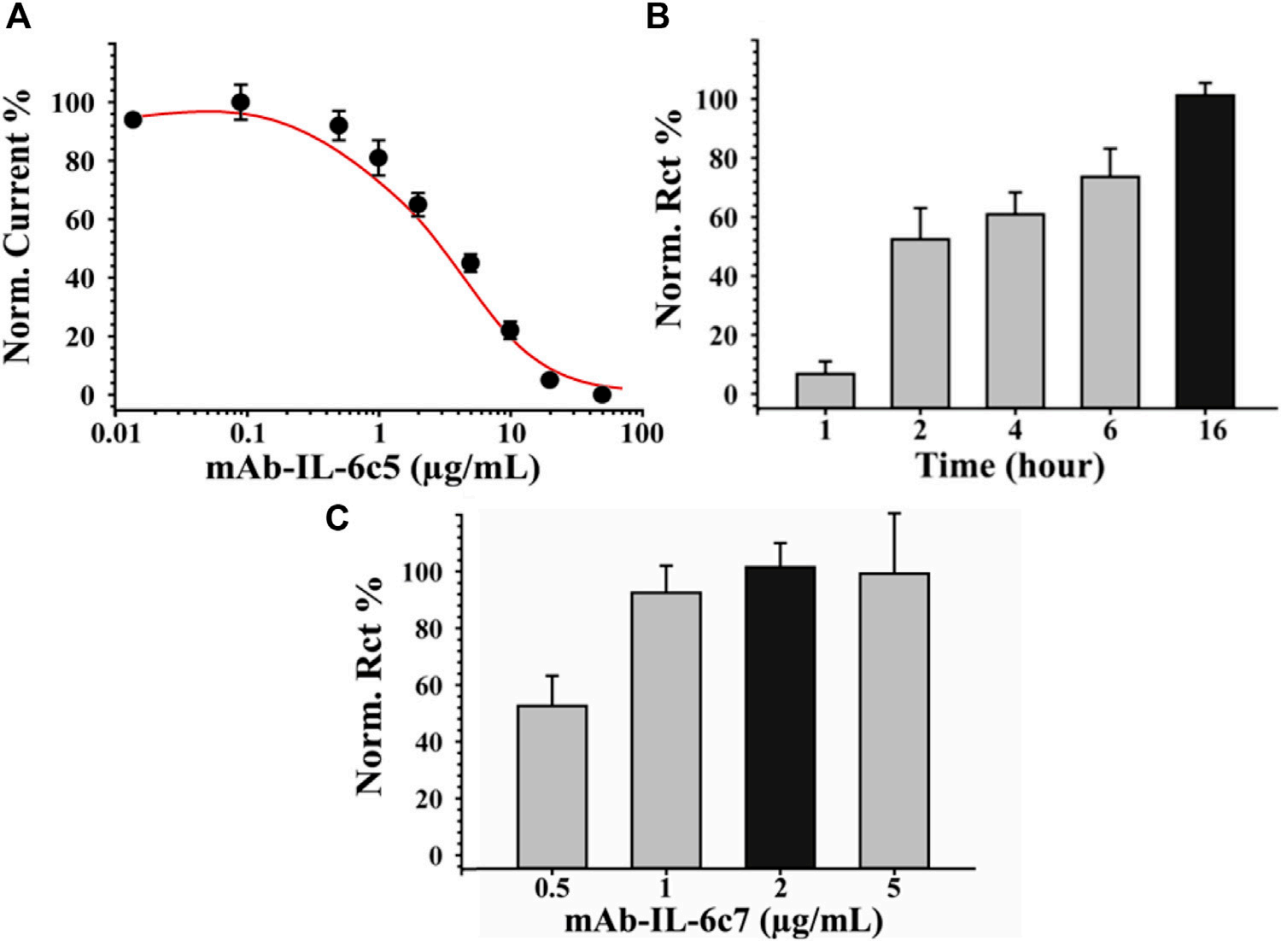
Optimization of Immunosensor fabrication. Panel **(A)** reports the binding curves obtained using different concentrations of mAb-IL-6c5 (from 0 to 50 μg/mL). Panel **(B)** and **(C)** report the optimization of the incubation time obtained using SPE-modified with biochar previously activated with EDC/NHS and of the mAb-IL-6 clone7 concentration, necessary to complete the sandwich assay, respectively. EIS was used as a sensing technique for all the experiments employing 0.010 M [Fe(CN)_6_]^4−/3−^ solution, in 0.05 M PBS +0.01 M KCl, pH 7.4 as the electroactive probe. The error bars represent the standard deviation computed using six different electrodes for each tested condition.

To fabricate a sensitive and reproducible label-free immunosensor, it is crucial to optimize the immunological chain constructions. For this purpose, various concentrations of primary antibody (0, 0.5, 1, 5, 10, 20 μg/mL) were investigated, and the relative results are shown in Figure 2A. Each of these steps was characterized impedimetrically using 10 mM [Fe(CN)_6_]^3-/4-^ as an electroactive probe. It can be seen the higher the antibody concentration, the higher the charge transfer resistance (Rct). This is likely due to the formation of a thicker layer of mAb-IL-6c5 (high density) that, having a large steric hindrance, considerably reduces the diffusion of the electrochemical probe to the electrode interface. The 70%–80% value of the response curve (Figure 2A), which corresponds to 1 μg/mL mAb-IL-6c5, defines the amount of antibody, which guarantees the highest sensitivity. This concentration of primary antibody is the best compromise between a too densely packed antibody film and a sparsely populated mAb-IL-6c5 surface, conditions that could impair the analytical performance of the immunosensor. Using 1 μg/mL of mAb-IL-6c5, the optimal incubation time (IT) for the covalent immobilization of the primary antibody was studied. Five different IT (0, 1, 2, 4, 16 h) have been tested. Figure 2B shows the normalized Rct as a function of time, indicating 16 h (overnight) as the best incubation time, both in terms of electrode covering and repeatability (RSD % = 5%). Therefore, it was selected as the IT clone-5 immobilization. The concentration of the second receptor probe (mAb-IL-6c7), required to complete the sandwich assay, was subsequently determined. Figure 2C reveals that 2 μg/mL of mAb-IL-6c7 is the optimal concentration in terms of covering effect (highest Rct) and reproducibility (RSD%<9%) for the sandwich formation (50 pg/mL of IL-6 were used for this experiment).

### 3.2 Electrochemical characterization of the layer-by-layer fabrication

**FIGURE 3:**
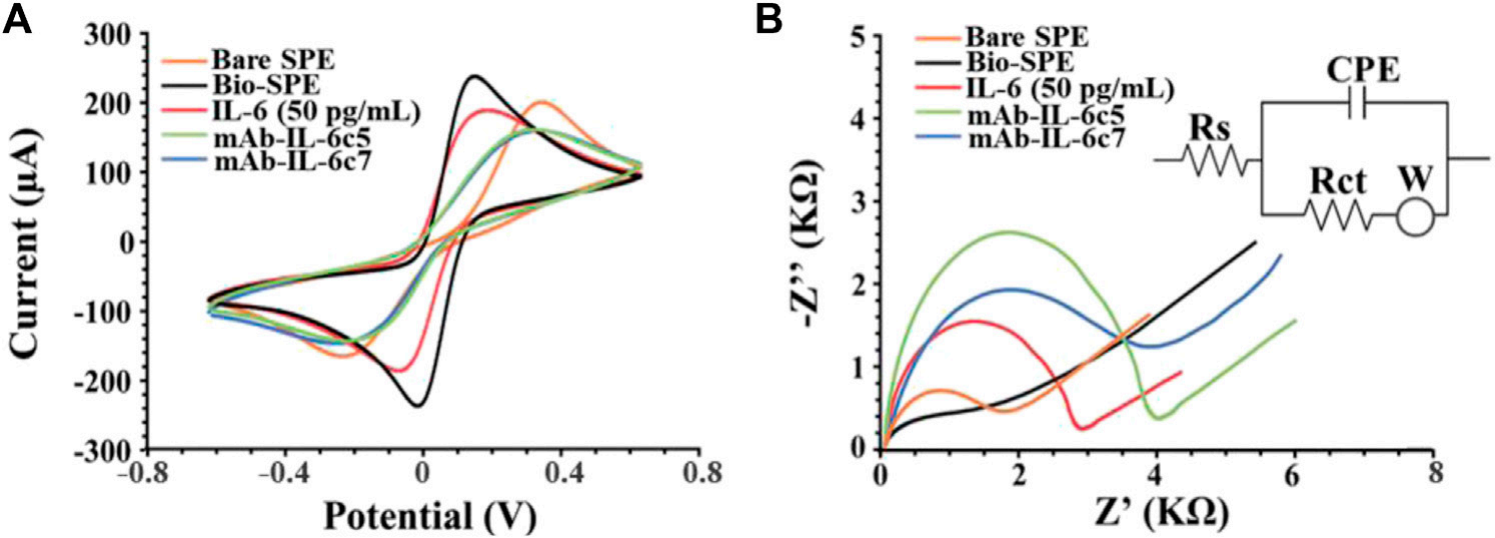
Electrochemical characterization of electrode interfaces during the multistep biosensor build-up. **(A)** Cyclic voltammograms and **(B)** Nyquist’s Plot recorded in 0.010 M [Fe(CN)_6_]^4−/3−^ in 0.05 M PBS (IL-6 50 pg/mL). Curves of one representative immunosensor of at least 3 analysed SPEs are presented. Randles model for the equivalent circuit (solution resistance (Rs), charge transfer resistance (Rct), the capacitance of double layer (CPE), and Warburg impedance (W)) is reported as inset in panel **(B)** (Barsoukov, 2018).

Once the fabrication protocol of the label-free immunosensor was optimized, Cyclic voltammetry (CV) and electrical impedance spectroscopy (EIS) were used as complementary techniques to comprehensively characterize the electrochemical behaviour of the electrode/electrolyte interface throughout each step of biosensor fabrication (detailed in Section 2.4 and Figure 1). CV’s electrochemical parameters such as anodic and cathodic peak current ratio (Ipa/Ipc), peak-to-peak separation (ΔE) and heterogeneous electron transfer constant (k^0^ and k^0’^, voltammetrically and impedimetrically calculated, respectively) were evaluated, as well as their variations based on the immunological chain construction. The same was performed by EIS. Nyquist plots consist of two portions: the linear (low frequencies) portion associated with electrochemical behaviour constrained by diffusion, and the semicircle (high frequencies) portion associated with electrochemical processes subject to electron transfer, where its diameter corresponds to Rct. Figures 3A, B (CV and EIS responses obtained using 0.01 M [Fe(CN)_6_]^4-/3-^ as an electroactive probe) depict the effects of sequential layer deposition on the enhanced conductivity of the biochar-modified electrode (Bio-SPE). The modification of bare SPEs by drop-casting with biochar (Bio-SPE), resulted in a dramatic improvement of the typically sluggish surface kinetics of unmodified platforms, as evidenced by the reduction of ΔE (from 0.48 V to 0.11 V) and the Rct (from 1.8 KΩ to 0.3 KΩ) and the correlated heterogeneous electron transfer constant (Table 1). This improvement is correlated to two distinct factors: the enhancement of the electron transfer process correlated to the nature of biochar, already demonstrated in our previous works, and the 3-fold increase in the electroactive surface area of the WE (A). After the immobilisation of mAb-IL-6c5 (1 μg/mL) on Bio-SPE, the electrode exhibits an increase in ΔE and Rct, indicating a successful immobilisation of the capture primary receptor. This is due to a more hindered electrode interface, which reduces the diffusivity of the electroactive probe and slows the electron transfer process. EIS and CV for this step show indications of the formation of a kinetic barrier to electron transfer of [Fe(CN)_6_]^4-/3-^. This isconsistent with the increased Rct recovered from the Randles equivalent circuit model inserted in Figure 3B. The Randles model for the equivalent circuit (solution resistance (Rs), charge transfer resistance (Rct), capacitance of double layer (CPE), and Warburg impedance (W)) represents each component at the WE interface and in the solution during the electrochemical reaction in the presence of the conducting electrolyte [Fe(CN)_6_]^4-/3-^ (Randviir and Banks, 2013; Barsoukov, 2018). The additions of IL-6 (50 pg/mL) and mAb-IL-6c7 (sandwich formation) protein layers were accompanied by further increases in ΔE and Rct, providing additional evidence of an increasing kinetic barrier to electron transfer as the electrode is progressively functionalized (Table 1).

**TABLE 1:** Peak-to-peak separation (ΔE), anodic and cathodic peak ratio (Ipa/Ipc), and voltammetric and impedimetric heterogeneous electron transfer rate constant (k^0^, k^0’^) estimated for SPE using CV and EIS in 0.005 M [Fe(CN)_6_]^4-/3-^, in PBS pH 7.4, during IL-6-immunosensor fabrication (mean values are based on three experiments).

	ΔE (V)	Ipa/Ipc	k^0^ 10^−4^ (cm/s)	Rct (kΩ)	k^0’^10^–3^ (cm/s)
**Bare SPE**	0.45 ± 0.03	1.2 ± 0.1	-	1.6 ± 0.1	23 ± 2
**Bio-SPE**	0.17 ± 0.02	0.99 ± 0.05	25 ± 2	0.6 ± 0.1	36 ± 2
**mAb-IL-6c5**	0.25 ± 0.03	1.3 ± 0.1	14 ± 1	3.2 ± 0.2	13 ± 1
**IL-6 (50 pg/mL)**	0.26 ± 0.03	1.3 ± 0.2	17 ± 1	2.2 ± 0.1	19 ± 1
**mAb-IL-6c7**	0.36 ± 0.03	1.3 ± 0.2	-	3.6 ± 0.3	8 ± 1

### 3.3 Characterization of the analytical performances of IL-6 immunosensor

**FIGURE 4:**
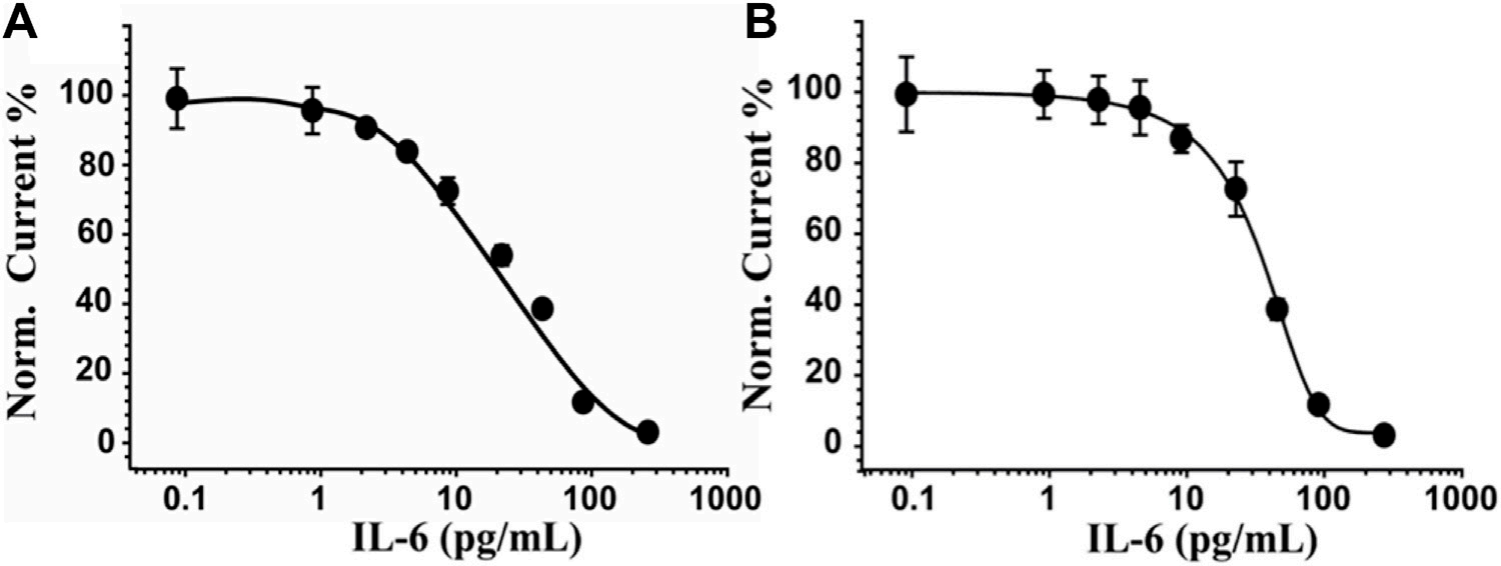
IL-6 sensor performance in PBS and serum-spiked samples. In **(A)** and **(B)** the dose-response curve obtained for PBS and in serum, respectively, by incubating concentrations of IL-6 ranging from 0 to 300 pg/mL and using SWV as a sensing technique and 0.010 M Fe(CN)_6_]^4−/3−^ in 0.05 M PBS as an electroactive probe. Curves of one representative immunosensor of at least 5 analysed SPEs are shown.

Under optimized conditions, the label-free electrochemical immunosensor was used to detect various concentrations of IL-6 (from 0 to 300 pg/mL) in buffer and serum samples. The sensing was performed by SWV using 0.010 M [Fe(CN)_6_]^4−/3−^ in 0.05 M PBS as an electroactive probe. The dose-response curve of the designed immunosensor for the detection of IL-6 in PBS (Figure 4A) shows a linear range from 2 to 250 pg/mL, a LOD of 0.78 pg/mL, a LOQ of 3.1 pg/mL and a reproducibility, calculated on six different SPEs, equal to 6%. Figure 4B depicts the relative results of the same experiment conducted on serum samples spiked with IL-6 concentrations ranging from 0 to 300 pg/mL and diluted 1:1 v/v in PBS. The label-free device’s sensitivity in a more complex matrix resulted in a LOD of 5.4 pg/mL, a LOQ of 7.1 pg/mL, and a linear range of 10–120 pg/mL. Reproducibility was marginally inferior (RSD% = 11%) respect to the analytical performance obtained in PBS, but comparable to other methods reported in the literature for the detection of IL-6 (see par. 3.6).

### 3.4 Cross-reactivity and storage stability

**FIGURE 5:**
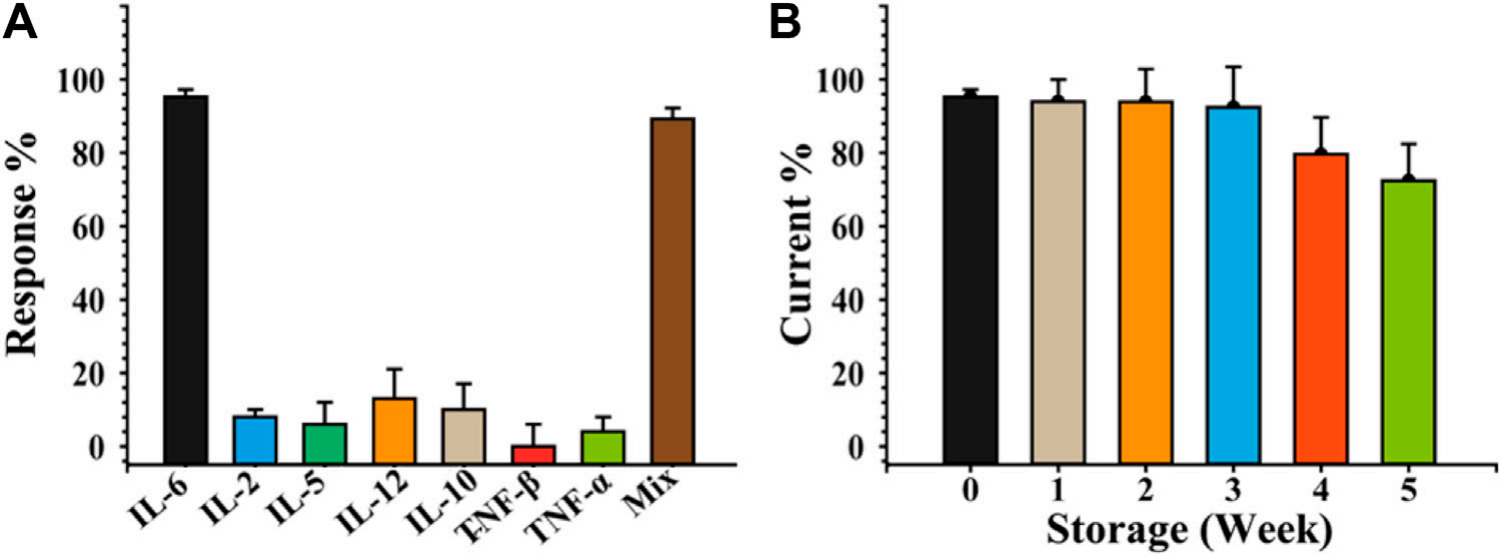
Selectivity and stability of the developed label-free sandwich-based IL-6 immunosensor. Immunosensor selectivity panel **(A)** and stability storing at 4 °C in a humid chamber, panel **(B)** study carried out using different cytokines as interferent agents. The sensing was performed by SWV by incubating 50 pg/mL of IL-6 and analysing 0.010 M [Fe(CN)_6_]^4−/3−^ in 0.05 M PBS. The error bars represent the standard deviation calculated using six different electrodes for each tested condition.

Due to their presence in serum following dermatitis, IL-2, IL-5, IL-10, IL-12, TNF-α, TNF-β, and a mixture of them with IL-6 (IL-2, IL-5, IL-10, IL-12, TNF-α, and TNF-βwere chosen as interferents model to test the selectivity of the IL-6 sensor. In this aim, the immunosensing devices were challenged individually with the above-mentioned cytokine at concentrations of 50 pg/mL (each one) in PBS. The SWV measurements were acquired under the same conditions used for IL-6 using six sensors for each cytokine. Figure 5A depicts the cross-reactivity, calculated as response percentage of the immunosensors (Eq. 8), concerning all the tested interferents, showing an high selectivity of the sandwich immunosensing format. Indeed, a percentage response always less than 20% was observed for each interferent species analysed, with 94% when IL-6 was mixed with all of them. This result is attributable to the use of a sandwich-format immunoassay in conjunction with two primary capture probes, mAb-IL-6c5 and c7, which favour the specific recognition of the target analyte while negating the interference effect of other cytokines. To evaluate the stability of the immunosensor under storage conditions, the response of IL-6-sensitive platforms was evaluated by storing the mAb-IL6c5 Bio-SPEs in PBS at 4 °C in a humid chamber for up to 5 weeks and assessing its ability to detect a concentration of IL-6 equal to 50 pg/mL. Figure 5B shows that the IL-6 sensor is stable (nearly constant response) for 3 weeks. Considering that no preservatives were added, a slight decline in performance was acceptable.

### 3.5 Sensing in human clinical samples

To validate the performances of our label-free electrochemical immunosensor, it was tested on up to sixteen sera from patients affected by PsO, and nine sera collected by no affected patients as negative controls. A commercial spectrophotometric ELISA kit was used to analyze the samples, and the electrochemical results were compared. The average and the standard deviation values of the three independent experiments were calculated (Table 2) with RE% as the relative percentage used as a measure of precision of the proposed method vs. ELISA test. The results deriving from the comparison between the two methods are quite satisfactory, showing a fair agreement when the IL-6 concentrations are above 10 pg/mL, while for lower concentrations and closer to the LOD of the immunosensor (5.4 pg/mL) there is an overestimation (samples n. 1, 7 and 11). Five subjects included in the study resulted with not determined IL-6 levels by the proposed tool. It is interesting to note how, even with the ELISA method, they all presented values equal to or lower than 4.3 pg/mL. Low serum IL-6 values can be determined by a low degree of disease (limited extension of the skin pathology), absence of psoriatic arthritis (associated with higher serum levels of IL-6) (Pietrzak et al., 2020), absence of cardiovascular comorbidities and recent disease onset. Future studies will be aimed at evaluating IL-6 levels in patients with PsO to determine whether the anatomical location, the extent or involvement of difficult sites (nails, scalp, genitals) and the intake of other drugs for comorbidities may determine a variation in IL-6 levels and a possible impact on the response to treatments. However, these results show how the proposed method is optimal for having correct monitoring of IL-6 in the blood in a short time and with a few microliters of sample and reagents in comparison with a spectrophotometric ELISA kit, which requests a minimum of 300 µL of blood samples and 180 min to perform the analysis.

**TABLE 2:** Comparison of IL-6 analysis results from human serum samples using two different methods, immunosensor and commercial spectrophotometric ELISA kit. * patient diagnosed with PsO, negative for IL-6 in both the assays.

Human serum samples	Immunosensor (pg/mL) (n = 3)	ELISA	RE%
		(pg/mL) (n = 3)	
**1**	9.7 ± 0.7	7.30 ± 0.01	33
**2**	23 ± 2	24.2 ± 0.1	−5
**3**	22 ± 2	24.2 ± 0.1	−9
**4**	n.d	4.3 ± 0.1	-
**5**	23 ± 1	24.20 ± 0.05	−5
**6**	n.d	4.3 ± 0.1	-
**7**	16.6 ± 0.1	15.05 ± 0.07	10
**8**	n.d	4.29 ± 0.07	-
**9**	n.d	4.22 ± 0.04	-
**10**	n.d	4.30 ± 0.01	-
**11**	8.1 ± 0.1	7.3 ± 0.1	11
**12**	15.3 ± 0.1	14.3 ± 0.1	7
13*	n.d	n.d	-
**14**	8.8 ± 0.1	7.29 ± 0.04	21
**15**	24 ± 2	24.22 ± 0.04	−1
**16**	15 ± 1	14.30 ± 0.01	−5
**(control sera, n = 9)**	n.d	n.d	-

^a^ d.: not detectable and under the sensitivity of the methods; 3 replicates for each sample (n = 3), **RE%** = [(Immunsensor-ELISA)/ELISA] x 100.

### 3.6 Comparison of biosensor performance (in serum) to earlier IL-6 biosensors reported in the literature

The analytical performances of the sandwich-based immunosensor here proposed were compared to a direct label-free immunosensor selective for IL-6 reported in the literature. The comparison was carried out by using the direct label-free immunosensor based on mAb-IL-6 clone 5, testing it in buffer solution and in serum samples. The summary of the results is reported in Table 3.

**TABLE 3:** Comparison of the analytical performance of electrochemical immunosensors developed over the past 3 years to reveal IL-6 in serum samples.

Device	LOD	Linear range	Ref
**IDEA/3-MPA/IL-6 mAb**	11.8 pg/mL	0.01–1 ng/mL	Oh et al. (2021)
**AuNPs-thionine-CMWCNTs**	3.87 pg/mL	10 to 0.8 ng/mL	Wang et al. (2020)
**ITO/AuNPb-PDOP**	3.4 pg/mL	5–1000 pg/mL	Crapnell et al. (2021)
Direct Immunosensor based on Bio-SPEs (our group)	5.4 pg/mL	30–138 pg/mL	Cancelliere et al. (2023a)
Sandwich Immunosensor based on Bio-SPEs	0.8 pg/mL	2–250 pg/mL	This work

Acronyms: IDEA (gold interdigitated electrode array), MPA (mercaptopropionic acid).

## 4 Conclusion

Active research is being conducted in the area of label-free electrochemical immunosensors for the detection of inflammatory molecules. Various electrode materials, sensing strategies, and signal amplification techniques have been explored to enhance the sensitivity, selectivity and stability of these devices. Efforts are currently being made to validate their performance by utilising clinical samples and comparing the results to standard laboratory techniques such as ELISA.

Herein, a sensitive, straightforward, and quick label-free immunosensor using disposable screen-printed electrodes for the measurement of IL-6 in human serum is reported. The analytical robustness of this device was tested in buffer solution, in spiked human serum samples and in twenty-five patients, revealing a minimal matrix effect and a satisfactory sensitivity and reproducibility of our label-free immunosensors comparable to a commercial ELISA kit. This result is even more remarkable if we consider the cost savings, speed, and capability to be performed by untrained personnel.

Nowadays, PsO is classified among Noncommunicable inflammatory skin diseases (ncISDs). ncISDs can be treated with novel and effective biological therapies, but diagnostic guidance on when and how to use them is still ambiguous. Ineffective and unguided treatments result in high socio-economic costs and waste of resources. It is, then, necessary to create treatments tailored to the patient based on the best drug choice and doses. The POC devices fit into this framework, which, for their speed and efficiency, can provide not only indications on which therapy to administer but, above all, to monitor the disease progress (Campione et al., 2021). According to the obtained results, our immunosensors can be considered a robust POC device for PsO and, due to its simplicity and adaptability, is suited to play an increasingly vital role shortly in enhancing healthcare.

## Data Availability

The original contributions presented in the study are included in the article/supplementary material, further inquiries can be directed to the corresponding authors.
